# ﻿A key to genera of Dikraneurini from China, with description of a new species of *Cornicola* Ohara & Hayashi (Hemiptera, Cicadellidae, Typhlocybinae)

**DOI:** 10.3897/zookeys.1145.94800

**Published:** 2023-02-06

**Authors:** Ye Xu, Christopher H. Dietrich, Dao-Zheng Qin

**Affiliations:** 1 Key Laboratory of Plant Protection Resources and Pest Management of the Ministry of Education, Entomological Museum, Northwest A&F University, Yangling, Shaanxi Province 712100, China Northwest A&F University Shaanxi China; 2 School of Agricultural Science, Jiangxi Agricultural University, Nanchang 330045, China Jiangxi Agricultural University Nanchang China; 3 Illinois Natural History Survey, Prairie Research Institute, University of Illinois, 1816 S. Oak St., Champaign, Illinois 61820, USA University of Illinois Champaign United States of America

**Keywords:** Auchenorrhyncha, distribution, Homoptera, leafhopper, morphology, polymorphism, taxonomy

## Abstract

The leafhopper genus *Cornicola* Ohara & Hayashi, previously recorded from Japan, is recorded from China for the first time and a new species, *C.maculatus* Xu, Dietrich & Qin, **sp. nov.**, is described and illustrated, including its color polymorphism. This genus has male genitalia and hind wing venation similar to those found in Empoascini but it is more appropriately placed in Dikraneurini. A key to species of *Cornicola* is given together with a key to the genera of Dikraneurini from China.

## ﻿Introduction

The tribe Dikraneurini is a diverse group and differs from other Typhlocybinae leafhoppers in lacking an appendix in the forewing and in usually having the hind wing submarginal vein complete and extended past vein RA or RP basad along the costal margin (Dietrich 2005). However, some genera included in this tribe either lack the hind wing submarginal vein (*Typhlocybella* Baker) or have this vein reduced or obsolete at the apex of the costal margin and thus resemble species of Empoascini (Viraktamath and Dietrich 2011; Dietrich 2013; Ohara and Hayashi 2022). One such genus in the latter category is *Cornicola* Ohara & Hayashi, 2022, with *C.mizuki* Ohara & Hyashi, from Japan, as its type species. In this paper, a second species of *Cornicola* is described as new from southwest China, together with a key to Chinese Dikraneurini genera. To date, Dikraneurini contain 74 genera and 497 valid species distributed throughout the world (Dmitriev et al. 2022) of which 25 genera and more than 60 species occur in China and have been studied by Matsumura (1931), Anufriev and Emeljanov (1988), Dworakowska (1972, 1979, 1993a), Chou and Ma (1981), Zhang and Chou (1988), Zhang (1990), Zhang and Kang (2007), Kang and Zhang (2012, 2013), Yang et al., (2012), Kang et al. (2013), Jiao and Yang (2015, 2020), Huang et al. (2018), Kang et al. (2018), Qin et al. (2020).

## ﻿Materials and methods

The specimens examined in this study were preserved in 95% ethanol stored for three years resulting in loss of the original color; they are now deposited in the insect collection of Illinois Natural History Survey, Champaign, Illinois (INHS). Morphological terminology used in this work follows Xu et al. (2021).

## ﻿Taxonomy

**Family Cicadellidae Latreille, 1825**

**Subfamily Typhlocybinae Kirschbaum, 1868**

### ﻿Tribe Dikraneurini McAtee, 1926

#### ﻿Genus Cornicola

Taxon classificationAnimaliaHemipteraCicadellidae

Ohara & Hayashi, 2022

E74BA9D3-CC05-57D0-BEB5-C4C8FFDFA76D

##### Type species.

*Cornicolamizuki* Ohara & Hayashi, 2022, by original designation.

##### Diagnosis.

*Cornicola* is easily distinguishable from all other known Typhlocybinae in having the following combination of characters: (1) crown of head much narrower than pronotum and strongly elevated above anterior margin of pronotum (Figs 3, 6); (2) forewing with vein R2 and RM arising from r cell and MCu from m cell (Fig. 9); (3) hind wing with submarginal vein obsolete along costal margin and anal vein branched (Fig. 10); (4) male pygofer with dorsal margin almost straight, with short preapical fingerlike process, folded mesad subapically, ventral appendage absent (Figs 16, 17); and (5) subgenital plates fused in proximal 1/3, with lateral macrosetal row (Fig. 19).

**Figures 1–15.**
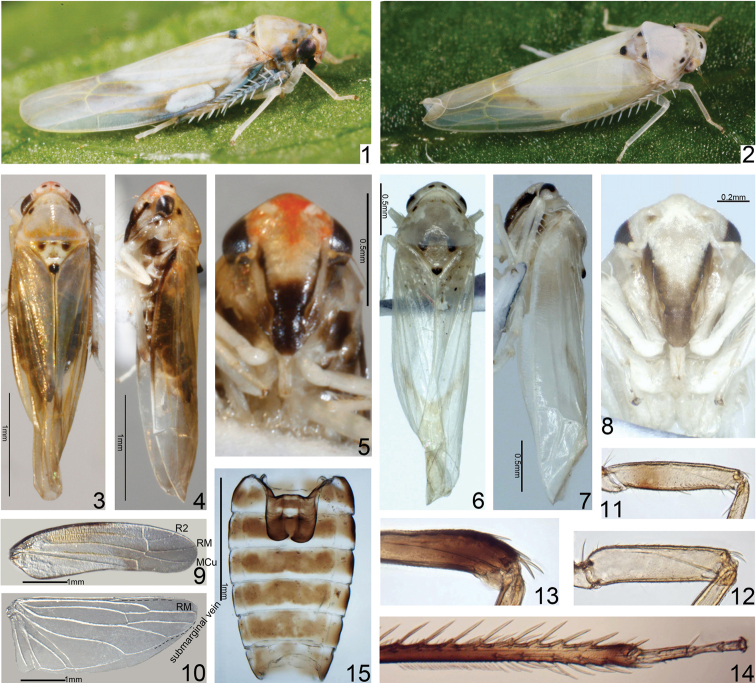
*Cornicolamaculatus* Xu, Dietrich & Qin sp. nov. **1, 2** Adults showing different body coloration **3** female adult, dorsal view **4** female adult, left lateral view **5, 8** face **6** male adult (abdomen removed), dorsal view **7** male adult (abdomen removed), left lateral view **9** forewing **10** hind wing **11** left femur and base of tibia, anterior view **12** left middle femur, anterior view **13** left hind femur apex and base of tibia, anterior view **14** distal part of hind tibia and tarsus, anterior view **15** sternal apodemes.

##### Notes.

Ohara and Hayashi (2022) recognized that *Cornicola* is related to *Igutettix* Matsumura, 1932 and therefore placed the genus in Dikraneurini; and also compared the genus to *Vilbasteana* Anufriev, 1970, *Koreoneura* Hossain & Kwon, 2021 and *Sweta* Viraktamath & Dietrich, 2011. However, the hind wing venation of *Cornicola* differs from the above-mentioned genera and instead resembles that of the Southeast Asian dikraneurine genera *Rakta* Dietrich, 2013 and *Albodikra* Dietrich, 2013 in having the submarginal vein obsolete or reduced apically along the costal margin of the hind wing (Fig. 10; fig. 2b, d in Dietrich 2013) and thus resembling that of Empoascini. *Cornicola* differs from these two genera in having an anteclypeus only slightly convex in both sexes (Figs 5, 8) (strongly swollen and broad in males of *Rakta* and *Albodikra*). Despite a strong resemblance of the hind wing venation of the new genus to the common pattern in Empoascini and some additional similarities in the male genitalia (e.g., elongate style), *Cornicola* is clearly more closely related to Dikraneurini and may represent a transitional form between Dikraneurini and Empoascini.

**Figures 16–24.**
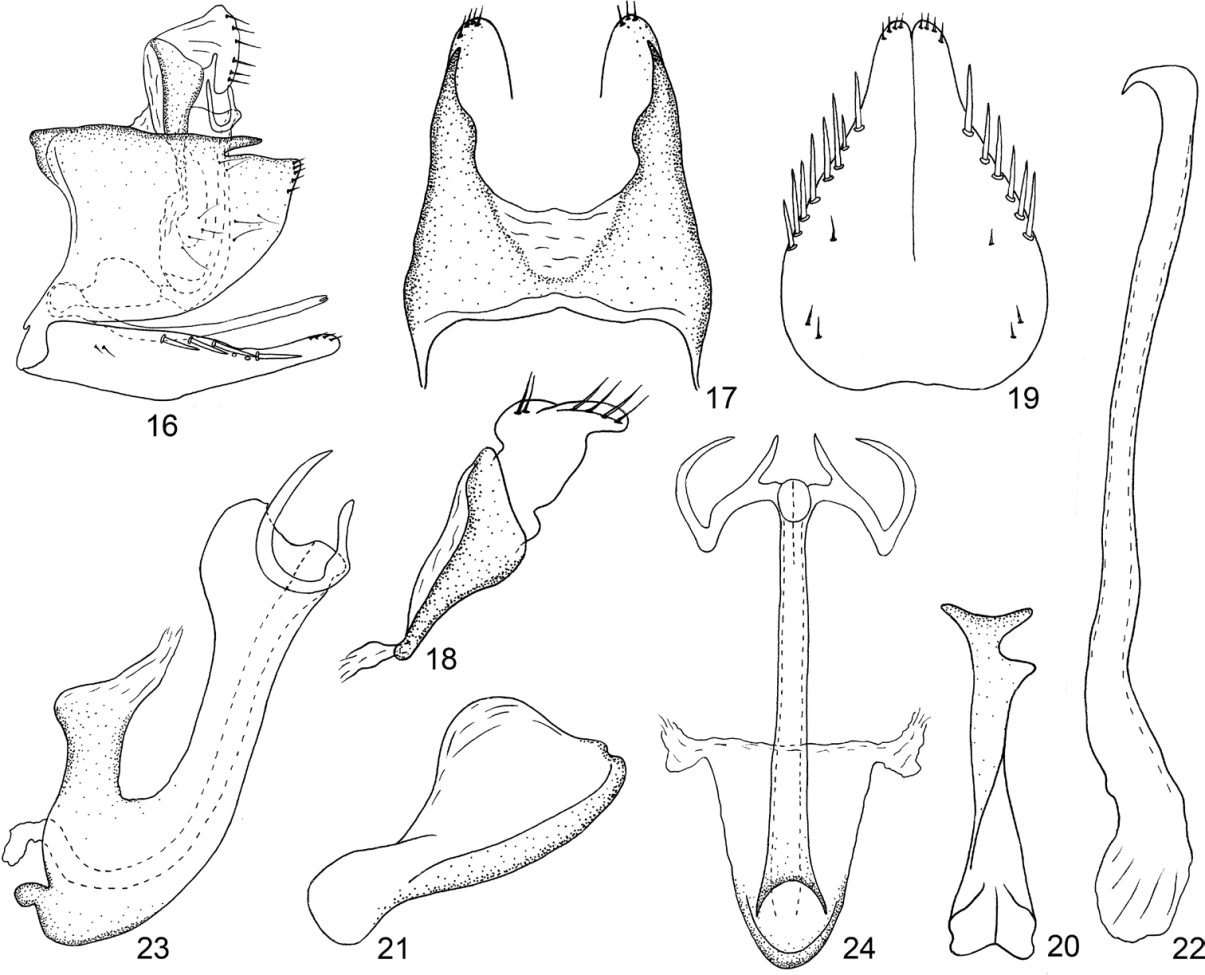
*Cornicolamaculatus* Xu, Dietrich & Qin sp. nov. **16** male genital capsule, left lateral view **17** male pygofer, dorsal view **18** anal tube, left lateral view **19** subgenital plates **20** connective, lateral view **21** connective, dorsal view **22** style **23** aedeagus, left lateral view **24** aedeagus, ventral view.

##### Distribution.

Japan (Hokkaido, Honshu, Shikoku) and China (Chongqing).

### ﻿Key to species of *Cornicola* Ohara & Hayashi (males)

**Table:** 

1	Male 2S apodemes extended nearly to posterior margin of segment V; aedeagus with shaft not widened at apex in lateral view, distal processes not forked at base, evenly curved in posterior view	***Cornicolamizuki* Ohara & Hayashi**
–	Male 2S apodemes reaching to end of segment IV (Fig. 15); aedeagus with shaft apex widened in lateral view (Fig. 23) distal processes forked near base, longer fork bent at acute angle in posterior view (Fig. 24)	***Cornicolamaculatus* Xu, Dietrich & Qin, sp. nov.**

#### ﻿ Cornicola maculatus

Taxon classificationAnimaliaHemipteraCicadellidae

Xu, Dietrich & Qin sp. nov.

645922AE-6AF9-50AA-A8AA-E0F474EF158E

https://zoobank.org/8BADF99F-7AA7-45AA-B06D-484E0D6565D0

Figs 1–15 , 16–24

##### Type materials.

***Holotype*.** ♂ (INHS) S.W. China, Chongqing Jinyan Shan, 29.829630°N, 106.376380°E, 872 m, 10 Sep. 2016, CH Dietrich, sweep roadside, CN-16-08. ***Paratypes*.** 4♂5♀(INHS) same data as holotype.

##### Description.

Body length: male 3.1–3.5 mm, female 3.2–3.5 mm.

Adults of this species are polymorphic with two color forms, one being mostly white (Figs 6, 7) and other with extensive reddish-brown coloration (Figs 3, 4) in both males and females (Figs 1–8). Reddish-brown morph (Figs 1, 3–5): Crown beige, with two small black oval patches and two irregular whitish patches surrounding dark red coronal suture, frontoclypeus with lateral dark brown band in lower half extending to base of antenna, lorum orange to dark (Figs 1, 3, 5). Eyes dark, ocelli irregularity with whitish spots (Figs 1, 3–5). Pronotum mostly orange red, with two small oval patches behind eyes, mesonotum with suborbicular spots, otherwise whitish except heart-shaped black patch medially in scutellum (Figs 1, 3–5). Forewing orange to reddish, veins brown; hind wing hyaline, veins white (Figs 1, 3, 4, 9). Front and middle legs almost hyaline, whitish except tarsus brown, hind legs brown (Fig. 1). White morph (Figs 2, 6–8): white overall with black spots and maculate, as in reddish-brown morph.

Basal sternal abdominal apodemes parallel sided, reaching end of segment IV (Fig. 15). Male pygofer almost triangular in lateral view, dorsal margin with fingerlike process arising near distal third of dorsal margin and extended posterad, not reaching apex; distal lobe bearing 6 or 7 microsetae, ventral margin with 8 or 9 feeble microsetae, dorsal bridge occupying more than one-third length of pygofer (Figs 16, 17). Anal tube gradually narrowed apically (Fig. 18). Subgenital plate longer than pygofer lobe in lateral view, broad basally, fused in basal two-thirds, tapered distally, apex rounded and strongly narrowing, with sparse scattered microsetae, 6–8 macrosetae arranged in single row along each dorsolateral margin near midlength (Fig. 19). Connective widest medially with subapical angular projection in lateral view, apical margin emarginate medially (Figs 20, 21). Style apodeme much shorter than apophysis, preapical lobe absent, without conspicuous setae, slightly broadened preapically, apex smooth, slightly broadened then tapered to hooklike tip, curved laterad (Fig. 22). Aedeagus with shaft broad at base, narrowed near middle and with broad dorsal distal lobe in lateral view; pair of slender distal processes extended laterad from adjacent gonopore, each with short dorsomedially directed spine and elbow-like bend near midlength with distal part curved dorsomesad in posterior view and anterodorsad in lateral view (Figs 23, 24).

##### Notes.

This new species differs from *Cornicolamizuki* by the characters noted in the key.

##### Distribution.

China (Chongqing).

##### Etymology.

The species name is derived from the Latin words ‘*maculatus*’, referring to the black spots on the crown and thorax.

### ﻿Key to genera of Chinese Dikraneurini

**Table:** 

1	Subgenital plates fused basally (Fig. 19)	**2**
–	Subgenital plates separate	**6**
2	Hind wing with submarginal vein obsolete along costal margin (Fig. 10)	***Cornicola* Ohara & Hayashi, 2022**
–	Hind wing with submarginal vein complete, extending along costal margin, around apex	**3**
3	Hind wing with MP and CuA fused for short distance	***Karachiota* Ahmed, 1969**
–	Hind wing with MP and CuA separate, connected by a short cross-vein (Fig. 10)	**4**
4	Forewing with veins R2 and RM confluent preapically	***Motschulskyia* Kirkaldy, 1905**
–	Forewing with veins R2 and RM separate, connected by a cross-vein	**5**
5	Connective about twice longer than wide	***Cuanta* Dworakowska, 1993**
–	Connective nearly as long as wide	***Platfusa* Dworakowska, 1993**
6	Hind wing with veins R and RA free, connected by a cross-vein	***Urvana* Dworakowska, 1993**
–	Hind wing with veins R and RA confluent distally	**7**
7	Forewing with veins R2, RM and MCu confluent preapically	**8**
–	Forewing with veins R2 and MCu separate preapically	**9**
8	Male pygofer with dense setae distally; 2S apodemes surpassing segment III	***Flatseta* Jiao & Yang, 2015**
–	Male pygofer without dense setae distally; 2S apodemes not reaching segment III	***Takagioma* Thapa, 1989**
9	Pygofer ventral appendage present	***Golwala* Dworakowska, 1993**
–	Pygofer ventral appendage absent	**10**
10	Style moderately long and thin, longer than subgenital plate	***Uniformus* Jiao & Yang, 2020**
–	Style shorter than subgenital plate	**11**
11	Forewing with vein MCu reduced, not extending to wing margin	**12**
–	Forewing with vein MCu complete	**13**
12	Subgenital plates triangular, narrowed apicad	***Naratettix* Matsumura, 1931**
–	Subgenital plates nearly oblong, truncated apicad	***Dicraneurula* Vilbaste, 1968**
13	Connective absent	***Forcipata* DeLong & Caldwell, 1942**
–	Connective present	**14**
14	Connective immovably attached or fused with base of aedeagus	**15**
–	Connective movably articulated with base of aedeagus	**16**
15	Male pygofer with upper appendage, without articulated caudal sclerite; subgenital plate with few macrosetae	***Togaricrania* Matsumura, 1931**
–	Male pygofer without upper appendage, with articulated caudal sclerite; subgenital plate without macrosetae	***Trifida* Thapa & Sohi, 1986**
16	Forewing with vein R2 and RM confluent preapically	**17**
–	Forewing with veins R2 and RM separate, connected by cross-vein	**21**
17	Aedeagus with pair of processes on shaft	**18**
–	Aedeagus without processes	**20**
18	Subgenital plate with more than eight macrosetae, arranged roughly in two rows	***Michalowskiya* Dworakowska, 1972**
–	Subgenital plate with fewer than five macrosetae, arranged in single row	**19**
19	Male pygofer nearly rectangular, without process; subgenital plate with few basal setae	***Iniesta* Dworakowska, 1993**
–	Male pygofer variable in shape, with process; subgenital plate without basal setae	***Anaka* Dworakowska & Viraktamath, 1975**
20	Aedeagus with gonopore apical, with circle of unpigmented cuticular outgrowths; male 2S apodemes surpassing segment V	***Uzeldikra* Dworakowska, 1971**
–	Aedeagus with gonopore not as above; male 2S apodemes surpassing segment IV	***Igutettix* Matsumura, 1932**
21	Subgenital plates not surpassing pygofer lobe	**22**
–	Subgenital plates surpassing pygofer lobe	**23**
22	Style with well-developed preapical lobe	***Dikraneura* Hardy, 1850**
–	Style without preapical lobe	***Ayubiana* Ahmed, 1969**
23	Aedeagus with dorsal apodeme absent	***Riyavaroa* Dworakowska, 1993**
–	Aedeagus with dorsal apodeme present	**24**
24	Aedeagus with paired shafts	***Notus* Fieber, 1866**
–	Aedeagus with single shaft	**25**
25	Male pygofer triangular; aedeagus without processes	***Wagneriala* Anufriev, 1970**
–	Male pygofer nearly round; aedeagus with few processes	***Erythria* Fieber, 1866**

## Supplementary Material

XML Treatment for Cornicola

XML Treatment for Cornicola maculatus
